# METTL14 contributes to the progression of nasopharyngeal carcinoma through regulating the stability of AOC1 mRNA

**DOI:** 10.1186/s41065-024-00317-z

**Published:** 2024-07-02

**Authors:** Changan Hu, Shengguan Song, Shanglong Zhao, Zhen Xue, Xiwen Zhu

**Affiliations:** Department of ENT & HN Surgery, Nanjing Lishui People’s Hospital, Zhongda Hospital Lishui Branch, Southeast University, No. 86 Songwen Road, Lishui District, Nanjing, China

**Keywords:** METTL14, AOC1, Nasopharyngeal carcinoma, Proliferation

## Abstract

**Background:**

Nasopharyngeal carcinoma (NPC) is a malignant epithelial tumor of the nasopharyngeal mucosa with a high incidence rate all over the world. Methyltransferase-like 14 (METTL14) is a major RNA N6-adenosine methyltransferase implicated in tumor progression by regulating RNA function. This study is designed to explore the biological function and mechanism of METTL14 in NPC.

**Methods:**

METTL14 and Amine oxidase copper containing 1 (AOC1) expression were detected by real-time quantitative polymerase chain reaction (RT-qPCR). The protein levels of METTL14, AOC1, Cyclin D1, B-cell lymphoma-2 (Bcl-2), and N-cadherin were measured using western blot. Cell proliferation, cycle progression, apoptosis, migration, and invasion were assessed using 5-ethynyl-2’-deoxyuridine (EdU), Colony formation, flow cytometry, wound scratch, and transwell assays. The interaction between METTL14 and AOC1 was verified using RNA immunoprecipitation (RIP), methylated RNA immunoprecipitation (MeRIP), and dual-luciferase reporter assays. The biological role of METTL14 on NPC tumor growth was examined by the xenograft tumor model *in vivo.*

**Results:**

METTL14 and AOC1 were highly expressed in NPC tissues and cells. Moreover, METTL14 knockdown might block NPC cell proliferation, migration, invasion, and induce cell apoptosis *in vitro*. In mechanism, METTL14 might enhance the stability of AOC1 mRNA via m6A methylation. METTL14 silencing might repress NPC tumor growth *in vivo.*

**Conclusion:**

METTL14 might boosted the development of NPC cells partly by regulating the stability of AOC1 mRNA, which provided a promising therapeutic target for NPC treatment.

## Highlights

1. METTL14 expression was upregulated in NPC tissues and cell lines.

2. METTL14 knockdown suppressed NPC cell growth and metastasis.

3. METTL14 improved the stability of AOC1 mRNA AOC1 via m6A methylation.

## Introduction

As an epithelial malignant tumor arising from the nasopharyngeal mucosal lining, nasopharyngeal carcinoma (NPC) has been recognized as a considerable public health burden in South China and Southeast Asia [1, 2]. Currently, genetic susceptibility, exposure to environmental factors, and Epstein-Barr virus infection might be responsible for the etiology of NPC [3]. Since NPC occurs in the nasopharynx, early symptoms are not obvious or specific, most sufferers have already developed neck lymph node metastasis when they are diagnosed [4, 5]. Despite substantial progress in operative resection, radiotherapy, and chemotherapy, effectiveness is still disheartening [6, 7]. Accordingly, exploring the mechanisms underlying NPC process is imperative for identifying more effective therapeutic targets.

Increasing studies are pointing toward that epigenetic modifications of the genome, reversible and inheritable processes modulate gene expression without altering the DNA sequence, which might be another pathway leading to the development of human cancers [8]. At present, a new research area of epigenetic modulation controlled by internal RNA modifications has gained extensive attention [9, 10]. Among them, N6-methyladenosine (m6A) modification represents one of the prevalent internal mRNA modifications among eukaryotes [11]. It can be installed by methyltransferase complexes (writers), containing methyltransferase-like 14 (METTL14), METTL3, and Wilms tumor 1-associated protein (WTAP) [12, 13]. Conversely, it is removed by m6A demethylases (erasers) consisting of obesity-associated protein (FTO) and alkylation repair homolog protein 5 (ALKBH5) [14]. Apart from that, the biological effects of m6A modification require the recruitment of reader proteins, such as YTH domain-containing family protein 1/2/3 (YTHDF1/2/3) and insulin-like growth factors 1/2/3 (IGF2BP1/2/3) [15]. Lately, emergent evidence has verified that imbalanced m6A modification might participate in regulating the pathogenesis of various human cancers via different RNA processing events, such as RNA stability and translational regulation [16, 17]. Interestingly, as the major m6A methyltransferase, METTL14 might provide an RNA-binding scaffold to facilitate RNA substrate recognition and activation, and to escalate the catalytic capability of METTL3, which catalyzes the conversion of adenosine (A) to m6A [18]. Furthermore, WTAP was found to be required for the accumulation of METTL3 and METTL14 in nuclear speckles [19]. In the field of tumor research, several studies have suggested that METTL3 and WTAP might boost NPC cell growth and metastasis via mediating m6A modification [20, 21]. However, there is currently no report about the underlying epigenetic regulation of METTL14 in the pathogenesis of NPC.

Nowadays, extensive laboratory work has shown that METTL14 might affect the stability or translation efficiency of mRNAs in an m6A-dependent way in a variety of tumors [22–24]. In this research, a public prediction server SRAMP exhibited that Amine oxidase copper containing 1 (AOC1), a copper-containing amine oxidase that catalyzes the deamination of polyamines, possesses multiple potential m6A sites. Beyond that, AOC1 was identified as the possible target of METTL14 in NPC cells. Therefore, in this project, we further inferred whether METTL14 might regulate NPC malignant progression by mediating AOC1 mRNA stability.

## Materials and methods

### Clinical samples and cell culture

After participants signed written informed consent, forty-seven freshly NPC and matched nasopharyngeal epithelial tissues were provided from the Nanjing Lishui People's Hospital. Subsequently, these specimens were immediately kept at a stable temperature of -80˚C. For this research, approval was obtained from the Ethics Committee of Nanjing Lishui People's Hospital.

Meanwhile, this project was authorized by the Ethics Committee of Nanjing Lishui People's Hospital.

Under a humidified atmosphere at 37˚C with 5% CO_2,_ human nasopharyngeal epithelial NP69 cell line (CL-0804, Procell, Wuhan, China) was cultured in a specialized medium (CM-0804, Procell), and two human NPC cell lines C666-1 (AW-CCH168, Abiowell, Changsha, China) and HNE-3 (TW-CL-04137, Shtongwei, Shanghai, China) were cultivated in RPMI-1640 medium (AW-MC002, Abiowell).

### RT-qPCR

In this experiment, total RNA was prepared based on the Trizol reagent (Invitrogen, Paisley Scotland, UK). After reversely transcribing 1 μg total RNA into cDNA using Prime Script RT Master Mix (Takara, Tokyo, Japan), amplification reaction was performed with SYBR Green PCR Kit (Takara). Finally, relative expression was normalized to β-actin and calculated by the 2^–ΔΔCt^ method. Primers used were shown in Table 1.

**Table 1:** The sequences of primers for RT-qPCR were presented

Name		Primers for PCR (5’-3’)
METTL14	Forward	GTAGCACAGACGGGGACTTC
	Reverse	TTGGTCCAACTGTGAGCCAG
AOC1	Forward	GCTGCGGACAACTTCAACTG
	Reverse	CGGTAGTGCACCAAGTGAGT
β-actin	Forward	CTTCGCGGGCGACGAT
	Reverse	CCACATAGGAATCCTTCTGACC

### Western blot assay

In short, total proteins were collected from tissues and cell lines with RIPA buffer (Keygen, Nanjing, China), followed by quantification with BCA protein assay kit. After being electrophoresed on 10% separating gel, samples were shifted onto PVDF membranes (Invitrogen), which were labeled at 4˚C with primary antibodies: METTL14 (ab220030), AOC1 (ab278497), Cyclin D1 (ab16663), B-cell lymphoma-2 (Bcl-2) (ab32124), N-cadherin (ab280375), and β-actin (ab8226). The next day, ECL reagent (Solarbio) and Image J software were used to detect the bands after incubation with secondary antibody for 2 h. Besides, these antibodies were provided by Abcam (Cambridge, MA, USA).

### Cell transfection

For METTL14 knockdown system, the short hairpin RNAs (shRNA) sequence of METTL14 was introduced into pLKO.1 lentiviral vector (Addgene, Cambridge, Massachusetts, USA) to generate sh-METTL14 lentivirus plasmid. Meanwhile, pLKO.1 empty vector was used as sh-NC. After being co-transfected into 293 T cells with these plasmids along with lentivirus package plasmid mixtures, cell supernatants were collected and inferred into NPC cell lines along with 8 µg/mL polybrene. At last, stably transfected cells were selected by using 5 µg/mL puromycin for 2 vector.

In addition, pcDNA vector specific to AOC1 (NM_001272072.2) and pcDNA empty vector GenePharma (Shanghai, China) were transfected into NPC cell lines using Lipofectamine 3000 (Invitrogen) for 48 h.

### Cell proliferation

For EdU assay, 4 × 10^3^ NPC cells were cultured in 96-well plates, followed by addition with fresh medium containing EdU working solution (20 μM, RiboBio, Guangzhou, China) for 2 h. After being fixed, sample from each group was permeabilized with 0.5% Triton-X-100 for 10 min. Subsequently, an Apollo reaction cocktail was added to these samples, which then were stained with DAPI for 30 min. Finally, a fluorescence microscope was applied for EdU-positive cells.

For colony formation experiment, 500 transfected NPC cells were allowed to grow for two weeks. Then, visible colonies were washed and fixed, followed by staining with 0.1% crystal violet. At last, the clones were imaged and quantified.

### Flow cytometry

Transfected NPC cells were subjected to 70% ethanol fixture and Propidium Iodide dyeing (PI, Bender Med System, Vienna, Austria) away from light. 20 min later, a flow cytometer was used to analyze cell percentage in each phase.

For cell apoptosis assay, the harvested NPC cells were re-suspended in binding buffer. Then, the cells from each group were subjected to 5 μL Annexin V-FITC and 10 μL PI double-staining under the dark environment. At last, apoptotic cells were quantified using a flow cytometer.

### Wound scratch and transwell invasion assays

NPC cells were cultured to reach 50–60% confluency. Then, a linear scratch wound was created using a sterile pipette tip (record 0 h) on the cell monolayer, followed by gentle washing. After being cultured for 24 h, images were acquired based on an inverted microscope.

In addition, NPC cell invasion ability was analyzed based on transwell chambers with Matrigel-precoated inserts (Chemicon, Temecula, CA, USA). In brief, NPC cells were introduced into upper chamber, whereas medium supplemented with 10% FBS was filled in bottom counterpart. 24 h later, invasive cells were immobilized, dyed, and counted using a microscope.

### RNA immunoprecipitation (RIP) assay

After being lysed in complete RIP lysis buffer (Millipore, Molsheim, France), the acquired C666-1 and HNE-3 cell extracts were incubated with magnetic protein A/G beads conjugated with anti-METTL14 or anti-IgG for 6 h at 4˚C. After digested, coprecipitated RNAs were isolated and detected using RT-qPCR assay.

### Methylated RNA immunoprecipitation (MeRIP)

The Magna MeRIP m6A kit (Millipore, Molsheim, France) was applied for this experiment. In short, total RNAs from C666-1 and HNE-3 cells transfected with sh-NC or sh-METTL14 were extracted using TRIzol (Invitrogen). After being fragmented to about 300 bp using Fragmentation Reagent (Invitrogen), one-tenth volume of fragmented RNA was aliquot for “Input”. Meanwhile, magnetic protein A/G beads were mixed with m6A antibody (MABE1006, Millipore) or anti-mouse IgG 9 (a negative control), which were incubated with the rest fragmented RNA. After being eluted and purified, methylated RNAs were subjected to RT-qPCR analysis.

### Dual-luciferase reporter assay

At first, the m6A sites of AOC1 mRNA were predicted based on an online bioinformatics software SRAMP and found enriched m6A peaks along the AOC1 sequence. Three m6A sites with high confidence scores in the CDS region of 1000- to 2500- nt were detected. Then, AOC1 CDS portion possessing 3 potential m6A sites was cloned into pmirGLO plasmid, generating wild-type (WT)-AOC1 construct. Meanwhile, m6A methylated site mutations of AOC1 were synthesized by Genscript (Nanjing, China) and then introduced into pmirGLO plasmid, namely as mutant (MUT)-AOC1 construct. After the co-transfection of these constructs and sh-NC or sh-METTL14 into C666-1 and HNE-3 cells, Reporter Assay System (Promega, Madison, WI, USA) was applied for the detection of the luciferase activity in cell lysates.

### Tumor xenograft assay

This animal experiment was authorized by the Animal Ethics Committee of the Nanjing Lishui People's Hospital. Briefly, 5–6 week-old male nude mice (Slaike Jingda Laboratory, Hunan, China) were randomly assigned to two groups (*n* = 5 for each group). Subsequently, 2 × 10^6^ C666-1 cells with sh-NC or sh-METTL14 were subcutaneously injected in the right of the back of mice under a specific pathogen-free environment. During this period, the size was measured according to a caliper on every three days basis. Twenty-four days after injection, excised tumors were subjected to image and weight after euthanized mice, followed by western blot and IHC staining analysis.

### Statistical analysis

In this research, GraphPad Prism7 software was used to analyze the data, which was expressed in the form of mean ± standard deviation (SD). Statistical significance was accepted for *P* < 0.05. Significance of two or more groups was completed using Student’s *t*-test or one-way analysis of variance (ANOVA) with Tukey’s tests.

## Results

### METTL14, an m6A-RNA methyltransferase, was elevated in NPC tissues and cells

**Fig. 1:**
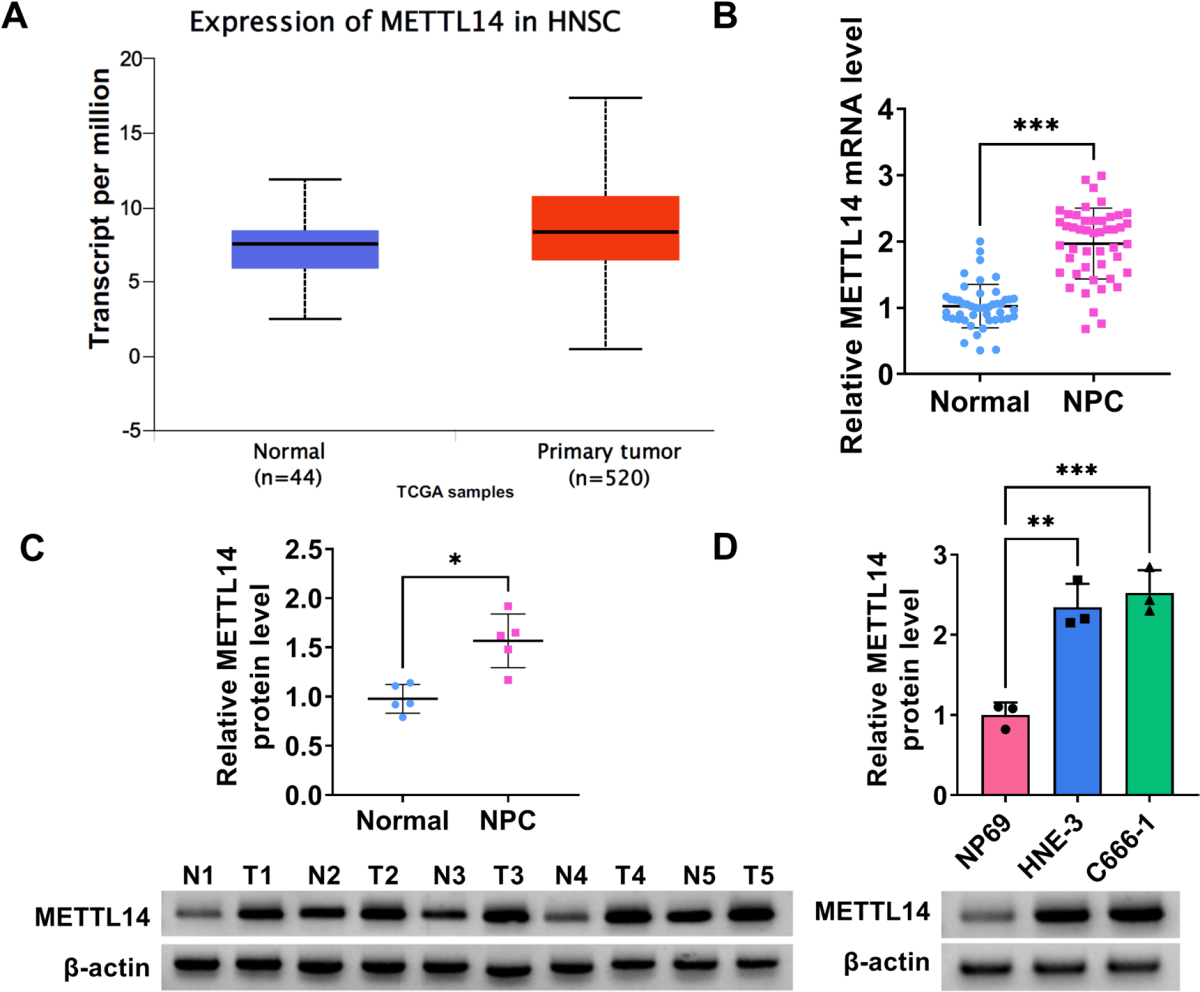
Expression of METTL14 in NPC tissues and cell lines. **A** UALCAN database presented the expression level of METTL14 in Head and Neck squamous cell carcinoma (HNSC) and normal samples. **B** RT-qPCR assay was applied to measure METTL14 mRNA level in 47 pairs of NPC tumor tissues and normal tissues. **C** Western blot analysis of METTL14 protein level in 5 NPC tumor tissues and 5 normal tissues. (**D**) METTL14 protein level in NP69, HNE-3, and C666-1 cells were determined using western blot. *P < 0.05, **P < 0.01, ***P < 0.001

To identify the functional role of m6A modification in NPC, METTL14, the core m6A methyltransferase was analyzed in TCGA samples based on the UALCAN database. As shown in Fig. 1A, METTL14 expression was apparently enhanced in Head and Neck squamous cell carcinoma (HNSC) relative to normal tissues. Moreover, we found that METTL14 mRNA level and protein level were highly expressed in NPC tumor tissues compared with their respective control groups (Fig. 1B and 1C). Beyond that, our data also validated the significant upregulation of METTL14 in NPC cell lines (HNE-3 and C666-1) versus NP69 cells (Fig. 1D). Together, these results indicated that the upregulation of METTL14 might be involved in NPC progression.

### Silencing of METTL14 might block NPC cell development *in vitro*

**Fig. 2:**
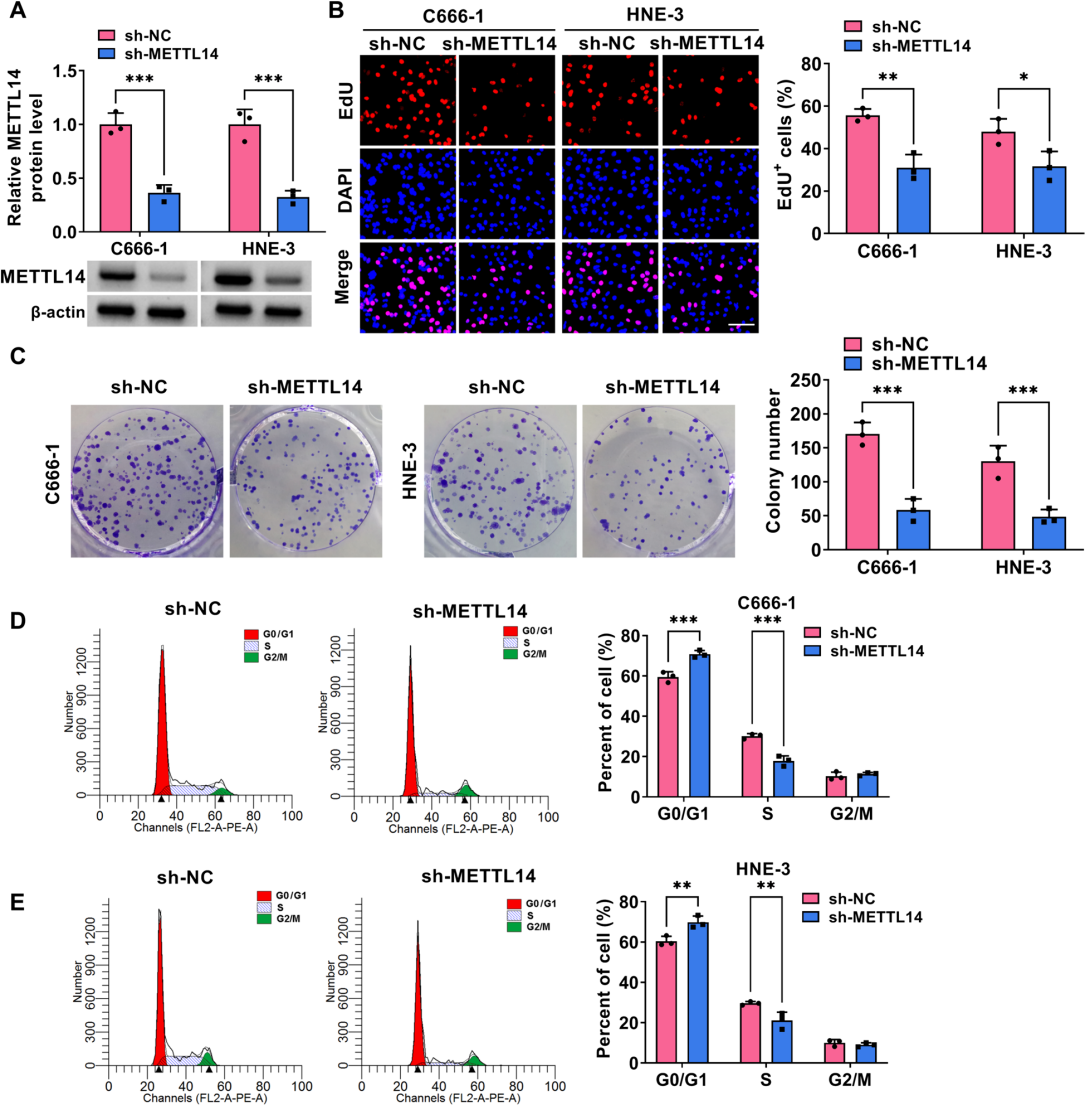
Effects of METTL14 downregulation on NPC cell proliferation. HNE-3 and C666-1 cells were transfected with sh-NC or sh-METTL14. **A** The knockdown efficiency of METTL14 was detected in NPC cells using western blot. **B** and **C** Cell proliferative ability was assessed in transfected NPC cells using EdU and colony formation assays. **D** and **E** Cell cycle progression was measured in transfected NPC cells using flow cytometry assay. *P < 0.05, **P < 0.01, ***P < 0.001

**Fig. 3:**
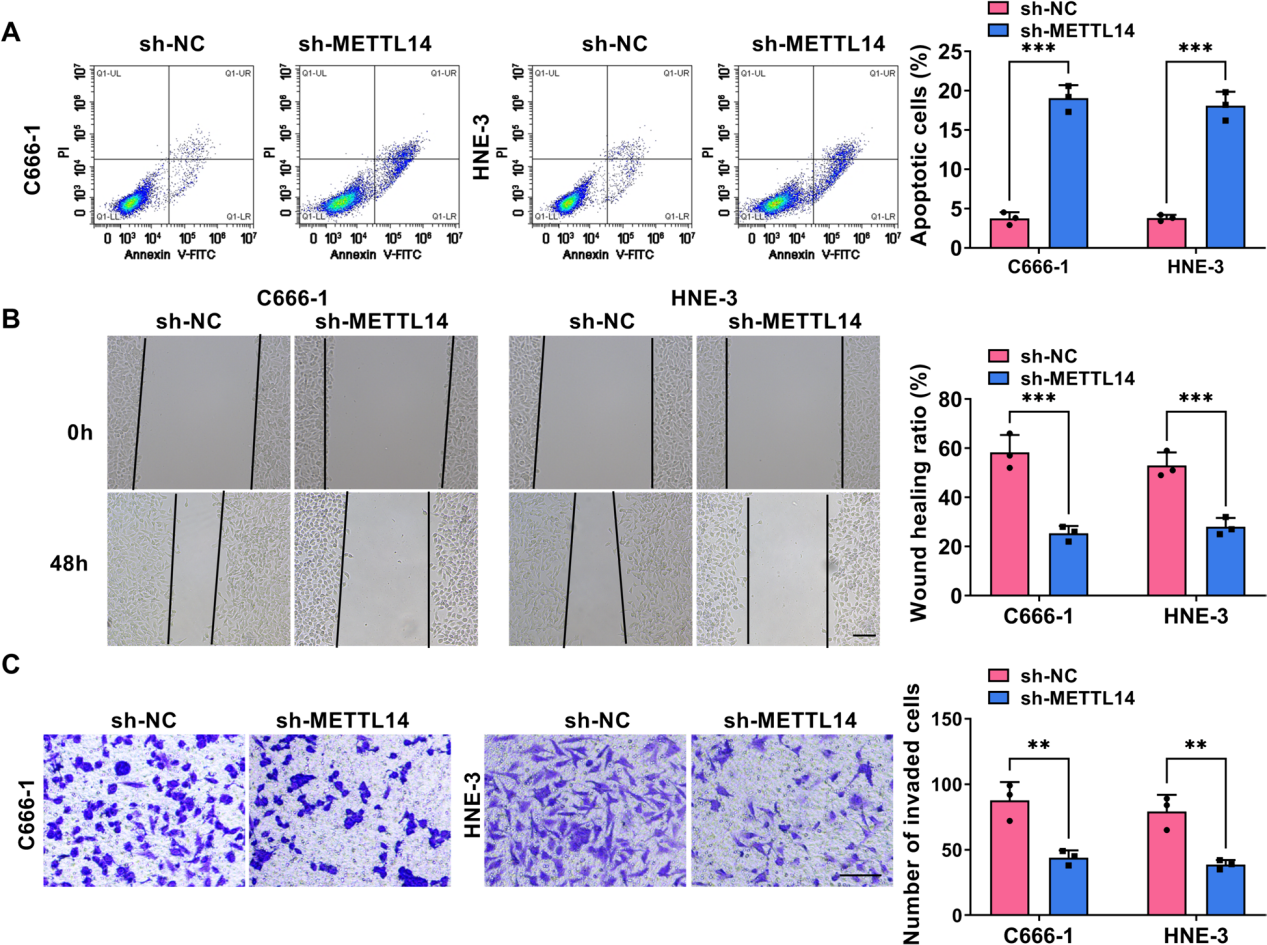
Influences of METTL14 knockdown on NPC cell apoptosis, migration, and invasion. HNE-3 and C666-1 cells were transfected with sh-NC or sh-METTL14. **A** Flow cytometry assay was used to assess apoptosis rate in transfected NPC cells. **B** Scratch assay was performed to examine cell migration ability was in transfected NPC cells. **C** Transwell assay was conducted to measure cell invasion in transfected NPC cells. *P < 0.05, **P < 0.01, ***P < 0.001

Then, to check whether METTL14 was critical to NPC cell growth and metastasis, *in vitro* loss-of-function analyses were carried out in HNE-3 and C666-1 cells. At first, western blot results displayed that METTL14 protein level was clearly reduced in sh-METTL14-transfected NPC cell lines in comparison with the control group (Fig. 2A). Functionally, reduced EdU-positive cell and colony numbers were viewed due to the downregulation of METTL14 in HNE-3 and C666-1 cells (Fig. 2B and 2C). Furthermore, flow cytometry assay displayed that the NPC cells in the sh-METTL14 group have an arrested cell cycle with a significant enhancement percentage of G0/G1-phase, and an obvious decrease of S-phase (Fig. 2D and 2E), inlying the repression of METTL14 absence on cell cycle progression. In parallel, the silencing of METTL14 elicited an apparent increase in HNE-3 and C666-1 cell apoptosis (Fig. 3A). In addition, Scratch assay and Transwell assay presented that the deficiency of METTL14 might clearly hinder the migration and invasion ability of HNE-3 and C666-1 cells (Fig. 3B and 3C). Overall, these results indicated that METTL14 downregulation might impede NPC cell growth and metastasis *in vitro*.

### METTL14 might regulate the stability of AOC1 mRNA through m6A modification

**Fig. 4:**
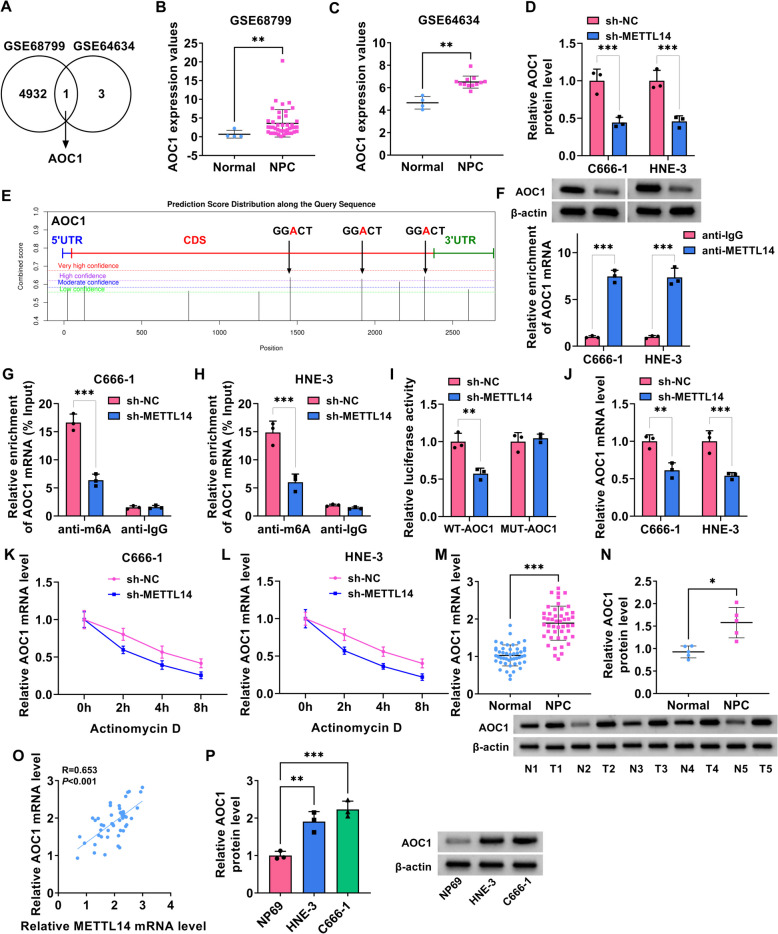
METTL14 might regulate the stability of AOC1 mRNA through m6A modification. **A** Venn diagram analysis of upregulated gene in NPC tissues in GSE68799 and GSE64634 datasets. **B** and **C** AOC1 expression in the GSE68799 and GSE64634 datasets. **D** Western blot analysis of AOC1 protein level in HNE-3 and C666-1 cells transfected with sh-NC or sh-METTL14. **E** SRAMP predicts that AOC1 mRNA has an m6A site. **F** Interaction between METTL14 and AOC1 was verified using RIP assay. **G** and **H** Changes in the m6A methylation level of AOC1 after inhibition of METTL14 were detected using MeRIP assay. **I** A luciferase reporter assay was used to analyze the binding between METTL14 and the AOC1. **J** AOC1 mRNA level was assessed in C666-1 and HNE-3 cells transfected with sh-NC or sh-METTL14 using RT-qPCR. **K** and **L** Effect of METTL14 knockdown on AOC1 mRNA stability after Actinomycin D treatment was measured using RT-qPCR in C666-1 and HNE-3 cells. **M** RT-qPCR analysis of AOC1 mRNA level in 47 pairs of NPC tumor tissues and normal tissues. **N** Western blot analysis of AOC1 protein level in 5 NPC tumor tissues and 5 normal tissues. **O** Pearson correlation analysis was applied to evaluate the expression association between METTL14 and AOC1 in NPC tissues. **P** AOC1 protein level was examined in NP69, HNE-3, and C666-1 cells using western blot. *P < 0.05, **P < 0.01, ***P < 0.001

To explore the molecular mechanism of METTL14 and check its downstream target in NPC, we screened highly expressed genes in NPC tissues from GSE68799 and GSE64634 datasets. As shown in Fig. 4A-C, AOC1 was the only candidate that was significantly upregulated candidate in these two data. Moreover, western blot assay found that the AOC1 protein level was clearly reduced in C666-1 and HNE-3 cells after sh-METTL14 introduction (Fig. 4D). Subsequently, to identify whether AOC1 downregulation in NPC cell lines was associated with m6A modification, a sequence-based m6A modification site predictor (SRAMP) was applied to predicate the potential m6A-modified sites of AOC1. As a result, we found enriched m6A peaks along the AOC1 sequence (Fig. 4E). Previous studies have suggested that METTL14 is a component of m6A methyltransferase complex, which is essential for the m6A catalytic process [25]. First of all, the interaction between METTL14 and AOC1 was confirmed in C666-1 and HNE-3 cells using RIP assay (Fig. 4F). Then, MeRIP assay was performed in C666-1 and HNE-3 cells and results exhibited that m6A modification level of AOC1 was distinctly decreased in METTL14 knockdown-transfected NPC cell lines (Fig. 4G and 4H). Consistently, dual-luciferase reporter analysis validated that the deficiency of METTL14 might significantly reduce the luciferase activity of AOC1 reporter vector in 293 T cells (F4g. 4I). Beyond that, RT-qPCR assay verified that AOC1 mRNA level was obviously blocked caused by METTL14 downregulation relative to the control group (Fig. 4J). In addition, actinomycin D assay showed that AOC1 mRNA stability was significantly diminished by METTL14 absence relative to the control group in C666-1 and HNE-3 cells (Fig. 4K and 4L). Apart from that, our data found a significant increase of AOC1 in NPC tissues compared with normal tissues (Fig. 4M and 4N). Meanwhile, the AOC1 mRNA level was positively correlated with METTL14 expression in NPC patients (Fig. 4O). Additionally, we further validated that AOC1 protein level was highly expressed in NPC cell lines (C666-1 and HNE-3) versus NP69 cells (Fig. 4P). In total, these results provided further evidence that METTL14 stabilizes AOC1 in an m6A-dependent manner in NPC cells.

### METTL14 knockdown might repress NPC cell growth and metastasis by interacting with AOC1

**Fig. 5:**
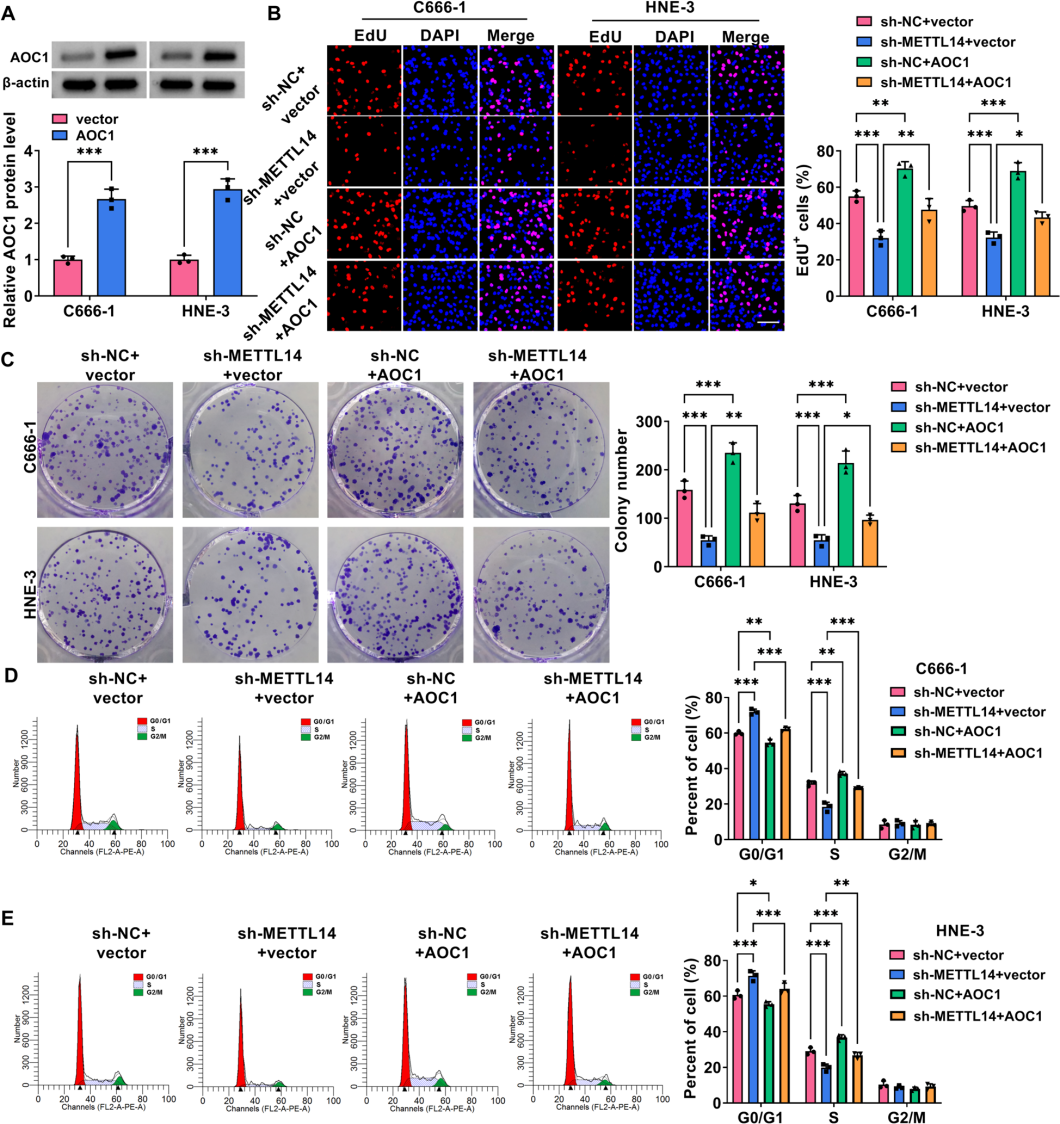
METTL14/AOC1 regulated NPC cell proliferation. **A** The overexpression efficiency of AOC1 in C666-1 and HNE-3 cells was analyzed using western blot. **B**-**E** HNE-3 and C666-1 cells were transfected with sh-NC + vector, sh-METTL14 + vector, sh-NC + AOC1, or sh-METTL14 + AOC1. **B** and **C** EdU and colony formation assays were conducted to measure cell proliferative ability in transfected NPC cells. **D** and **E** Flow cytometry assay was carried out to assess cell cycle progression in transfected NPC cells. *P < 0.05, **P < 0.01, ***P < 0.001

**Fig. 6:**
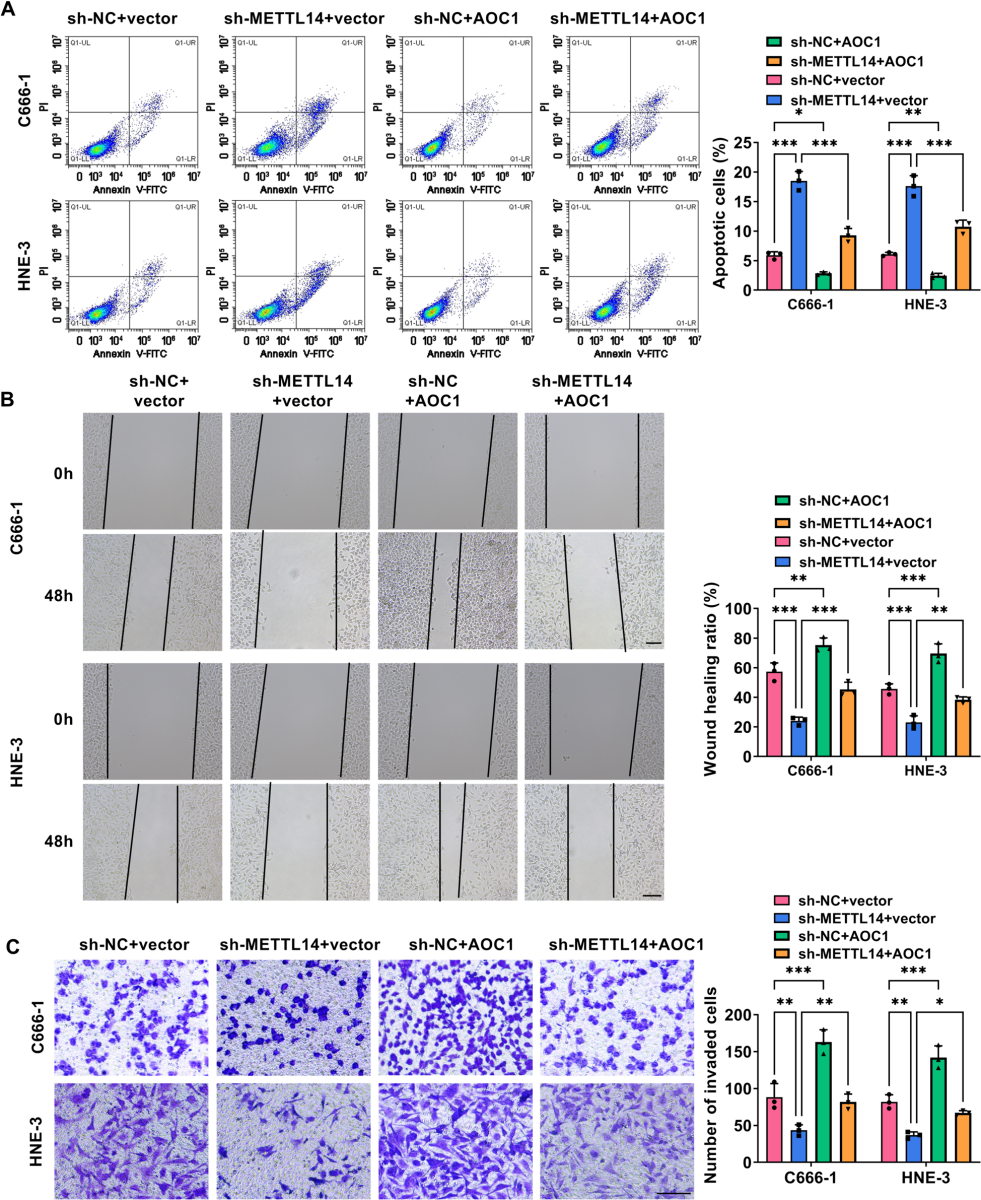
METTL14 modulated NPC cell apoptosis, migration, and invasion via interacting with AOC1. C666-1 and HNE-3 cells were transfected with sh-NC + vector, sh-METTL14 + vector, sh-NC + AOC1, or sh-METTL14 + AOC1. **A** Flow cytometry assay analysis of apoptosis rate in transfected NPC cells. **B** and **C** Scratch and Transwell analysis of cell migration and invasion ability was in transfected NPC cells. *P < 0.05, **P < 0.01, ***P < 0.001

**Fig. 7:**
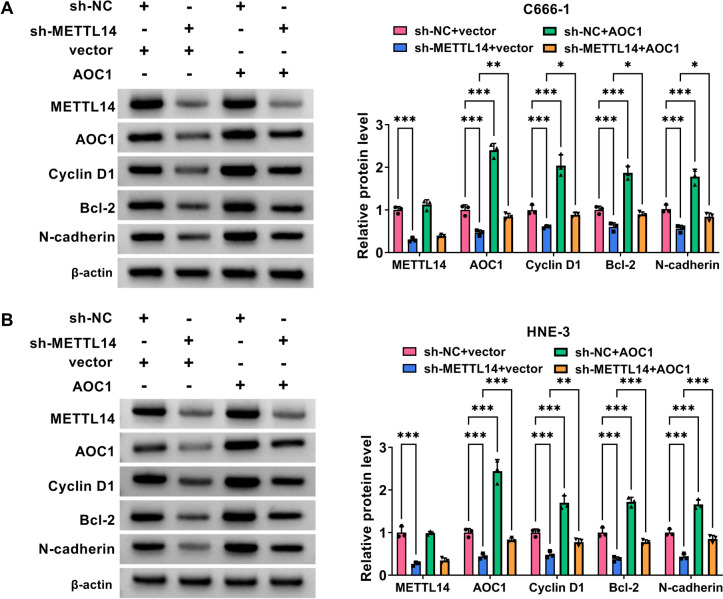
METTL14/AOC1 affected NPC cell proliferation, apoptosis, and migration-related markers. **A** and **B** METTL14, AOC1, Cyclin D1, Bcl-2, and N-cadherin protein levels were determined in C666-1 and HNE-3 cells transfected with sh-NC + vector, sh-METTL14 + vector, sh-NC + AOC1, or sh-METTL14 + AOC1 using western blot. *P < 0.05, **P < 0.01, ***P < 0.001

Subsequently, to investigate whether AOC1 mediated METTL14-dependent NPC growth and development, rescue assays were performed in C666-1 and HNE-3 cells. At first, western blot assay exhibited that AOC1 protein level was remarkably enhanced in AOC1-transfected NPC cell lines (Fig. 5A), implying that the overexpression efficiency of AOC1 is available. After that, functional analysis displayed that METTL14 deficiency-induced C666-1 and HNE-3 cell proliferative ability (Fig. 5B and 5C) and cycle progression (Fig. 5D and 5E) repression were partially abrogated by AOC1 overexpression. Simultaneously, METTL14 absence might promote C666-1 and HNE-3 cell apoptosis, which was effectively attenuated through AOC1 co-transfection (Fig. 6A). Furthermore, AOC1 upregulation might greatly ameliorate the inhibitory effects of METTL14 downregulation on C666-1 and HNE-3 cell migration and invasion (Fig. 6B and 6C). Similar to the above results, western blot assay presented that METTL14 silencing might markedly constrain the protein levels of Cyclin D1 (proliferation-related factor), Bcl-2 (anti-apoptosis marker), and N-cadherin (migration-associated factor) in C666-1 and HNE-3 cells, whereas these effects were partially abolished by AOC1 upregulation (Fig. 7A and 7B). Altogether, the above-mentioned results elucidated that AOC1 might alleviate the repression of METTL14 downregulation on NPC growth and metastasis *in vitro*.

### Silencing METTL14 might block NPC tumor growth *in vivo*

**Fig. 8:**
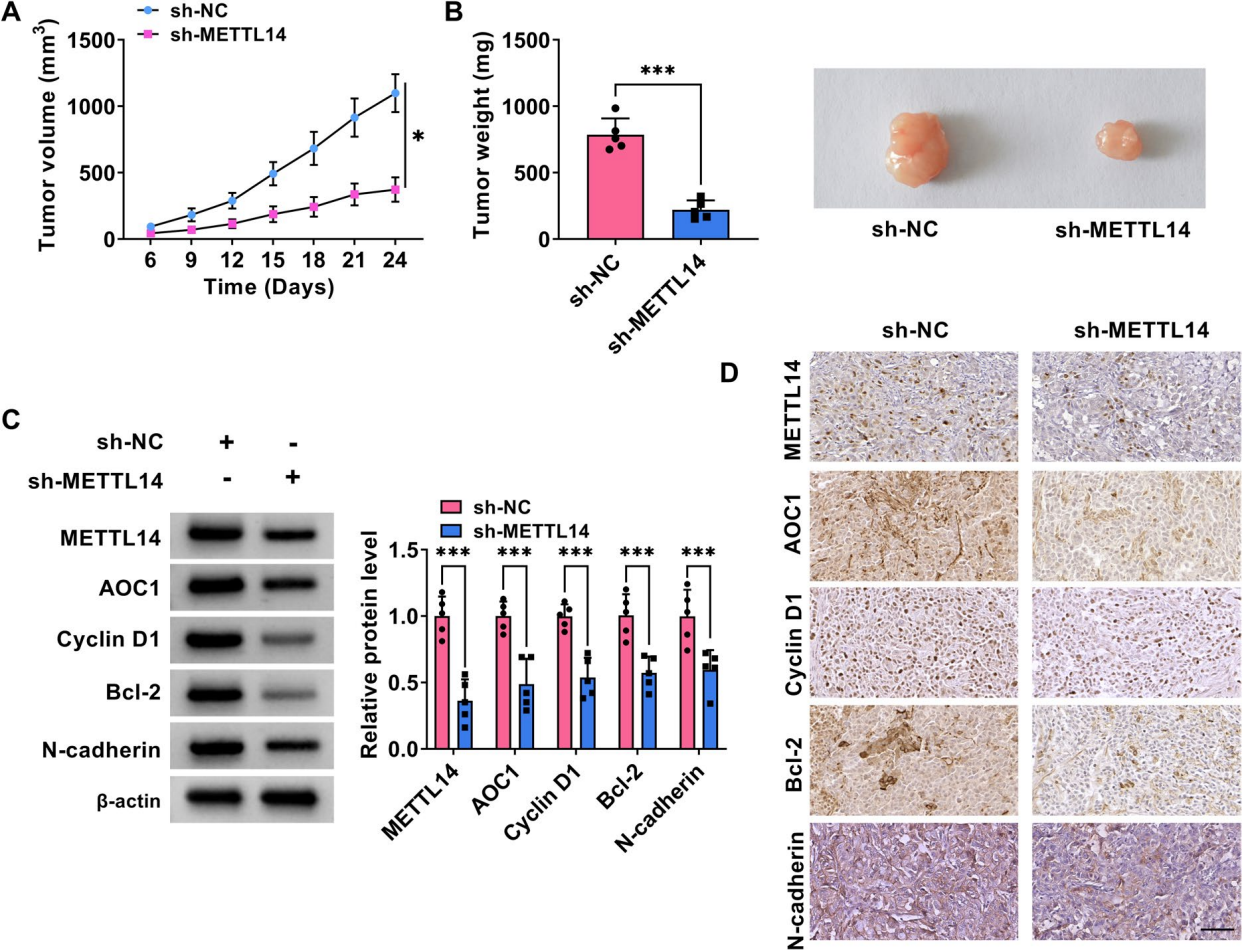
METTL14 absence might dampen C666-1 cell growth in a xenograft model. C666-1 cells with sh-METTL14 or sh-NC were inoculated subcutaneously into the nude mice. **A** Growth curve of xenografted tumors was presented. **B** Weight of resected tumor masses was shown. **C** Protein levels of METTL14, AOC1, Cyclin D1, Bcl-2, and N-cadherin in the xenografts were detected using western blot. **D** METTL14, AOC1, Cyclin D1, Bcl-2, and N-cadherin expression was gauged in xenografts using IHC staining. *P < 0.05, **P < 0.01, ***P < 0.001

Additionally, a xenograft tumor mouse model was established to validate the data about the repression of METTL14 knockdown on NPC cell malignancy *in vivo.* Results showed that the volumes and weights of in situ tumors dropped in response to METTL14 downregulation relative to those of the sh-NC control group (Fig. 8A and 8B), suggesting the inhibitory role of METTL14 deficiency on NPC tumor growth. Beyond that, western blot analysis exhibited that the protein levels of METTL14, AOC1, Cyclin D1, Bcl-2, and N-cadherin were clearly lower in tumor tissues derived from sh-METTL14-transfected C666-1 cells (Fig. 8C). In addition, IHC staining also found that the positive expression rate of METTL14, AOC1, Cyclin D1, Bcl-2, and N-cadherin was apparently declined in the sh-METTL14 group compared with the sh-NC groups (Fig. 8D). Accordingly, it is concluded that METTL14 deficiency could dampen NPC growth *in vivo*.

## Discussion

There are investigations indicating that aberrant epigenetic modification may occur at early stages of neoplastic development and perform an essential player in the pathogenesis of several human cancers [26]. As a new layer of epigenetic modulation, m6A RNA modification has gained substantial attention as a biochemical process that regulates cell growth and differentiation by controlling RNA splicing, stability, and translation [27, 28]. It has been reported that m6A modification is catalyzed via a multicomponent methyltransferase complex consisting of METTL3, METTL14, and WTAP, which is responsible for the major determination of m6A levels [29]. As an inactivated methyltransferase, METTL14 might act as a key adapter for METTL3 activity to increase methyltransferase activity through recognizing RNA substrates and methyl localization [25, 30]. Moreover, WTAP is a regulatory subunit whose function is to recruit METTL3 and METTL14 into nuclear speckles (correlated with target mRNA export) [19]. Of note, as a central component of the m6A methylated transferase complex, METTL14 has been found to be dysregulated and partake in different tumor development [24, 31]. Interestingly, METTL3 and WTAP have been shown to be upregulated and implicated in NPC cell proliferation and invasion [21, 32]. Nevertheless, few researchers have focused on the role of METTL14 in NPC progression. Herein, a significant upregulation of METTL14 in NPC tissues and cell lines was identified for the first time. Beyond that, functional analysis revealed the repression of METTL14 deficiency on NPC cell proliferation and metastasis *in vitro*. Consistently, *in vivo* experiments also discovered that METTL14 knockdown might hinder C666-1 cell growth in this research. On the whole, these results provided first-hand evidence that METTL14 might be an oncogene during NPC processes.

Regarding the molecular mechanism, METTL14 has been verified to participate in tumorigenesis by m6A-dependent modification of mRNA or non-coding RNA [23, 24, 31]. In the current work, AOC1, a secreted glycoprotein, was demonstrated as a probable downstream target of METTL14-mediated m6A modification in NPC cells. Furthermore, METTL14 was found to mediate m6A demethylation of AOC1 mRNA. METTL14 silencing might reduce AOC1 protein expression and mRNA stability in NPC cells.

As a copper-containing amine oxidase, AOC1 is responsible for catalyzing the deamination of polyamines to generate reactive oxygen species [33]. It has been reported that amine oxidases are closely linked to tumor growth and progression [34]. For example, positive expression of AOC1 was significantly associated with worse clinical outcomes in colorectal cancer and accelerated tumor cell aggressive phenotypes via inducing EMT pathway [35]. Meanwhile, AOC1 has been documented as an oncogene in gastric cancer through activating the AKT signaling pathway [36]. In terms of NPC, the overexpression of AOC1 might expedite tumor cell proliferation and migration [37]. Consistent with these findings, AOC1 expression was clearly increased in NPC tissues and cell lines in this study. Then, to further explore whether AOC1 is a major downstream target of METTL14 in modulating NPC development, rescue assays were performed. As expected, AOC1 upregulation might partially overturn METTL14 deletion-mediated NPC cell growth and metastasis inhibition. These observations further validated that m6A modification induced by METTL14 participates in regulating AOC1 in NPC via maintaining AOC1 mRNA stability.

## Conclusion

**Fig. 9:**
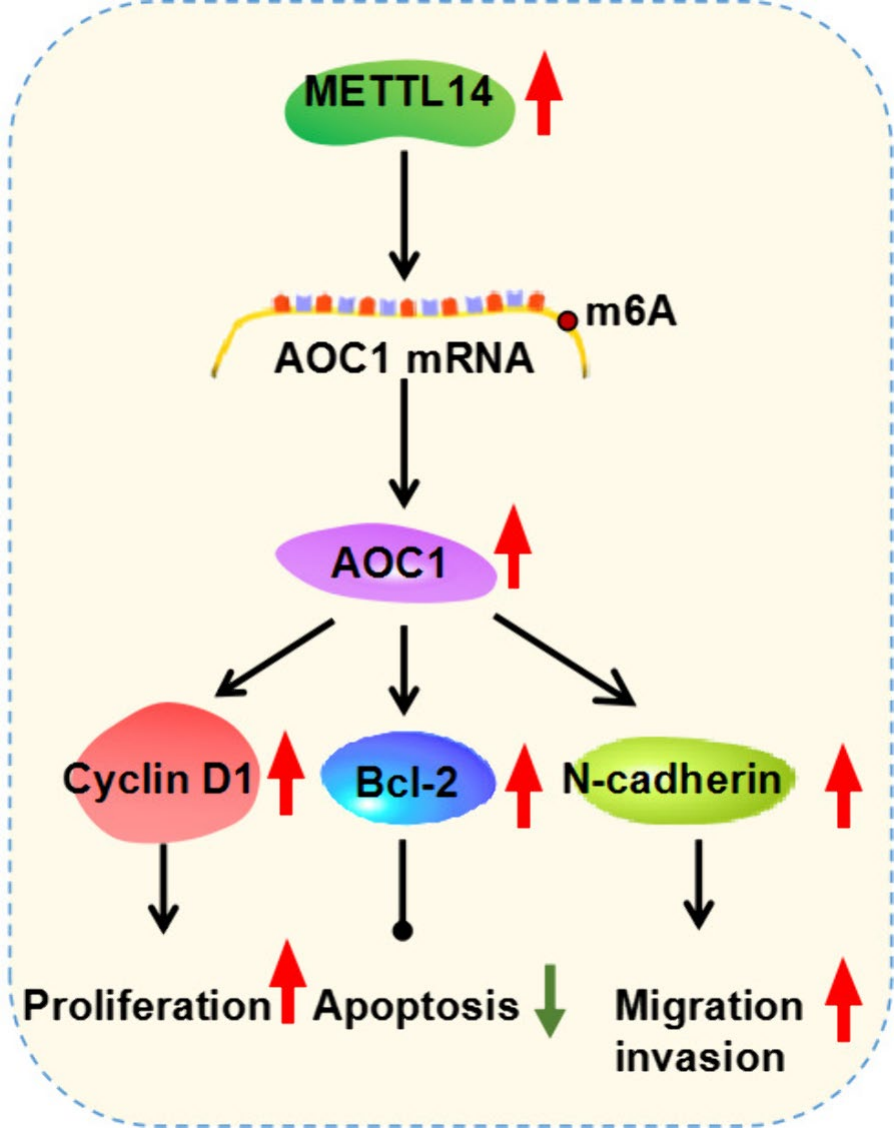
METTL14 promoted AOC1 expression via m6A modification and facilitated NPC progression

In summary, our present work elucidated compelling evidence that METTL14, an important m6A methyltransferase, was upregulated in NPC patients and cells. Moreover, METTL14 might facilitate NPC progression by increasing the stability of AOC1 mRNA (Fig. 9), providing a novel avenue of therapy for NPC.

## Data Availability

The analyzed data sets generated during the present study are available from the corresponding author on reasonable request.
